# Gamma activity in Autism Spectrum Disorder: Enhanced response to visual input

**DOI:** 10.1192/j.eurpsy.2024.653

**Published:** 2024-08-27

**Authors:** B. Kakuszi, B. Szuromi, M. Tóth, I. Bitter, P. Czobor

**Affiliations:** ^1^Psychiatry and Psychotherapy, Semmelweis University; ^2^OMIII–Nyírő Gyula Hospital, Budapest, Hungary

## Abstract

**Introduction:**

Autism spectrum disorder (ASD) is a childhood onset neurodevelopmental condition, that leads to permanent disability in a high proportion of cases. ASD is associated with a heterogeneous symptom presentation, which - besides social interaction and communication difficulties - encompasses altered sensory reactivity, including excessive hyper-sensitivity to stimuli, especially in the visual domain. Meta-analyses of fMRI studies revealed increased reactivity in visual task conditions in the temporal and occipital brain regions. Neural oscillations in the EEG gamma band are viewed as a candidate neurobiological marker for higher order sensory and perceptual processes, and social interactions.

**Objectives:**

We investigated changes in gamma activity in the EEG in the eyes open (EO) vs. eyes closed (EC) condition in order to identify the neurobiological underpinning of the enhanced sensitivity to visual input in ASD as compared to typically developing (TD) subjects.

**Methods:**

EEGs were obtained in EC and EO condition in ASD (N=23) and TD subjects (N=24) in an ongoing study. For EEG recording we used a high-density 128-channel BioSemi system, with 0.5 Hz frequency resolution. The spectral power in the gamma band (30-100Hz) was quantified by the power spectral density. To investigate whether changes in the gamma band were linked to changes in arousal instead of enhanced visual processing, we also examined alterations in the alpha band (8-13Hz) in the EO condition. Spectral power changes were determined for each EEG channel by computing the difference between the EC and EO conditions (EO-EC).

**Results:**

Spectral power in the gamma band showed changes in the opposite direction in the two study groups: ASD subjects manifested significant (p<0.05) increase, while TD subjects had a decrease in the EO vs. EC condition in the temporal and occipital brain regions. By contrast, the changes in the alpha band were similar, with both groups exhibiting a spectral power decrease in the EO compared to the EC condition.

**Conclusions:**

In ASD, an enhancement of gamma activity is present in the EO as compared to the EC condition in the posterior brain areas. These brain areas are involved in the processing of visual information, and gamma activity is considered as a measure of perceptual processes. Thus, the gamma alterations in the EO vs. EC condition may underlie the hyper-sensitivity symptoms to visual stimuli in ASD, and EEG can offer a simple to use tool to delineate the neurobiological foundation of the symptom presentation.

Funding statement: Hungarian Brain Research program,#NAP2022-I-4/2022

**Disclosure of Interest:**

None Declared

